# Captive gibbons (Hylobatidae) use different referential cues in an object-choice task: insights into lesser ape cognition and manual laterality

**DOI:** 10.7717/peerj.5348

**Published:** 2018-08-06

**Authors:** Kai R. Caspar, Larissa Mader, Fabian Pallasdies, Miriam Lindenmeier, Sabine Begall

**Affiliations:** 1 Department of General Zoology, Faculty of Biology, University of Duisburg-Essen, Essen, Germany; 2 Czech University of Agriculture, Department of Game Management and Wildlife Biology, Prague, Czech Republic

**Keywords:** Lesser apes, Object-choice task, Primate cognition, Laterality, Handedness

## Abstract

**Background:**

Utilization of visual referential cues by non-human primates is a subject of constant scientific interest. However, only few primate species, mostly great apes, have been studied thoroughly in that regard, rendering the understanding of phylogenetic influences on the underlying cognitive patterns difficult.

**Methods:**

We tested six species of captive gibbons in an object-choice task (*n* = 11) for their ability to interpret two different pointing gestures, a combination of body orientation and gaze direction as well as glancing as referential cues. Hand preferences were tested in the object-choice task and in a bimanual tube task (*n* = 18).

**Results:**

We found positive responses to all signals except for the glancing cue at the individual as well as at the group level. The gibbons’ success rates partially exceed results reported for great apes in comparable tests and appear to be similarly influenced by prior exposure to human communicative cues. Hand preferences exhibited by the gibbons in the object-choice task as well as in a bimanual tube task suggest that crested gibbons (*Nomascus* sp.) are strongly lateralized at individual but not at population level for tasks involving object manipulation.

**Discussion:**

Based on the available data, it can be assumed that the cognitive foundations to utilize different visual cues essential to human communication are conserved in extant hominoids and can be traced back at least to the common ancestor of great and lesser apes. However, future studies have to further investigate how the social environment of gibbons influences their ability to exploit referential signals. Gibbons’ manual laterality patterns appear to differ in several aspects from the situation found in great apes. While not extensive enough to allow for general conclusions about the evolution of hand preferences in gibbons or apes in general, our results add to the expanding knowledge on manual lateralization in the Hylobatidae.

## Introduction

Cognition of gibbons or lesser apes (Hylobatidae) has only sparsely been investigated. A number of studies have discussed different aspects of gibbon cognition, such as problem solving (e.g., Boutan, 1913), tool use (Cunningham, Anderson & Mootnick, 2006; Geissmann, 2009), comprehension of object permanence (Fedor et al., 2008; Anderson, 2012) and mirror-induced behaviour (e.g., Ujhelyi et al., 2000; Suddendorf & Collier-Baker, 2009). Despite that, our current understanding of the matter is still remarkably limited and respective research on the Hylobatidae lags behind compared to other primate groups, especially great apes (Hominidae) (Abordo, 1976; Cunningham, 2006). Due to small sample sizes and often pronounced idiosyncracies of the tested subjects, the results of many investigations remain inconclusive.

Since gibbons form the extant sister group to the great apes, knowledge on the cognitive psychology of this taxon can decisively contribute to the reconstruction of the mental abilities of the common ancestor of all hominoids. It might therefore eventually also promote to elucidate the origins of human cognition.

A cognitive trait with an insufficiently understood evolutionary history is the comprehension of referential gestures (Liebal & Call, 2012). By executing a referential signal, such as directional pointing, a sender intentionally guides the attention of a receiver to a specific point in space. This can be done in a declarative way, to solely reference to the position of an object, or in an imperative one in order to pose a request (Lyn, Russell & Hopkins, 2010). For humans, referential communication is of exceptional importance. Human infants comprehend and use declarative gestures to reference to the position of objects from an age of around one year on (e.g., Behne, Carpenter & Tomasello, 2005; Butterworth & Morissette, 1996) and might be able to interpret glance cues as referential signals at an even earlier age (Senju, Csibra & Johnson, 2008). In non-human primates, the presence of a similar usage and understanding of gestural as well as gaze (head and eye orientation) and glance (exclusively eye orientation) cues has long been debated (Leavens, Bard & Hopkins, 2017). Today it is evident that all genera of great apes can produce and comprehend declarative gestures such as pointing during intraspecific encounters and interspecific communication with humans (Leavens & Hopkins, 1998), although observations on the use of referential gestures in the wild are currently restricted to the genus *Pan* (Veà & Sabater-Pi, 1998; Pika & Mitani, 2006; Genty & Zuberbühler, 2014; Douglas & Moscovice, 2015). While gaze following in varying degrees of complexity has been demonstrated for most major primate taxa (e.g., lemurs: Ruiz et al., 2009; New World monkeys: Amici et al., 2009; Old World monkeys: Emery et al., 1997; gibbons: Liebal & Kaminski, 2012; great apes: Lurz et al., 2018), the capacity to comprehend gaze direction and glancing immediately as an intentional referential signal is usually attributed to hominids exclusively (Itakura & Tanaka, 1998; Barth, Reaux & Povinelli, 2005). For gibbons, despite their diverse repertoire of social gestures (e.g., Liebal, Pika & Tomasello, 2004) and famously complex vocal communication (Clarke, Reichard & Zuberbühler, 2006; Terleph, Malaivijitnond & Reichard, 2018), declarative non-vocal signalling has never been reported and their abilities to comprehend respective gestures as well as gaze and glancing cues remain largely unexplored.

A common method to study the comprehension of visual referential cues such as gestures and gaze direction by animals is the object-choice task (OCT). It requires a subject to use experimenter-given referential signals to correctly choose from a selection of objects to obtain a reward. Although most prominently applied to primates, OCTs have also been conducted with diverse other species, among them dogs, dolphins, flying foxes and corvid birds (Mulcahy & Hedge, 2012). Great apes perform in OCTs with variable success. Repeated tests of the same great ape species in OCTs often yield contradictory results, which likely reflect influences of rearing history and individual psychocognitive predispositions (Byrnit, 2009). In general, any type of referential signals mentioned so far appear to be problematic to interpret spontaneously, that is without prior experience. Nevertheless, it can be robustly stated that all hominid genera are in principal able to use most aforementioned cues reliably and appear to do so more proficiently than other primates such as Old World and New World monkeys (Itakura & Tanaka, 1998). Only the use of glance cues by gorillas has not been demonstrated yet (Peignot & Anderson, 1999), in contrast to the findings in orang-utans and chimpanzees (Itakura & Tanaka, 1998). Importantly, the experimental setup has been shown to profoundly influence the apes’ performances in the task (Barth, Reaux & Povinelli, 2005; Mulcahy & Call, 2009; Mulcahy & Suddendorf, 2011). Apes consistently perform notably better in OCTs with peripheral object presentation, compared to central object presentation (Mulcahy & Hedge, 2012). Despite that, the latter is used far more frequently in studies featuring primates.

Great apes that experienced prolonged close human contact performed better in the tests compared to those that did not, regardless of their species; this observation has been related to the “enculturation effect” (Call & Tomasello, 1994; Lyn, Russell & Hopkins, 2010). Hominids lacking pronounced exposure to human communicative signals usually do not respond above chance level when confronted with pointing gestures, gaze or glance cues. Enculturation evidently enhances the comprehension of these human communicative traits as well as diverse other behavioural patterns in chimpanzees, gorillas and orang-utans, being the most intensively studied primates in this regard (Call & Tomasello, 1996).

Although enculturation effects on cognition have provoked noticeable attention in the study of great apes, they have, to our knowledge, never been systematically investigated in gibbons. However, a remarkable report on how enculturation might influence cognitive processes in hylobatids was given by Inoue, Inoue & Itakura (2004) who tested a juvenile white-handed gibbon (*Hylobates lar*) that was raised in close contact to humans in an OCT. The gibbon was provided with different experimenter-given cues to locate food items hidden beneath one of two opaque cups. The cue types included pointing gestures, a combination of gaze and body orientation and glancing. The gibbon was able to interpret all given cues correctly without prior training and with a remarkable precision, rivalling the responses of enculturated great apes in comparable tasks. As the authors conclude, the gibbon’s performance likely was profoundly influenced by previous experience with human communicative cues which was transferred to the test situation by the subject. Of special interest is its apparent comprehension of glancing as a referential cue, a capacity that has been thought to be absent in non-hominid primates and that is only rarely observed in great apes (e.g., Itakura & Tanaka, 1998). None of the experimenter-given cues tested by Inoue, Inoue & Itakura (2004) are known to have any relevance to gibbons in an intraspecific context. Therefore, it can be assumed that gibbons being able to interpret such cues have adapted their behaviour towards the humans they interact with, demonstrating a high degree of flexibility regarding comprehension of communicative signals. In an additional OCT, conducted with seven captive white-handed gibbons, these remarkable results were not replicated (Yocom, 2010). Instead, the tested individuals consistently failed to use pointing and gaze cues to locate hidden rewards. However, only limited information on the methodology and subjects were given by the author, hindering the interpretation of results.

In the current study, we adapted the OCT protocol of Inoue, Inoue & Itakura (2004), and tested if the remarkable ethological plasticity of their subject is a general aspect of gibbon behaviour by focusing on zoo-housed hylobatids that experienced varying degrees of human contact. Since no gibbon in our sample could be classified as enculturated (see ‘Methods’ below), we predicted that none of the subjects would reach the overall success rate of 87.5% of the *H. lar* tested by Inoue, Inoue & Itakura (2004). However, the gibbons tested in our study showed varying degrees of human interaction (see below, Materials and Methods, *Subjects*). We, therefore, hypothesized that those subjects that experienced closer contact to humans would perform notably better than others. With regard to the presented referential signals, we hypothesized a decrease in success rates from near pointing to far pointing to body and gaze direction and eventually to glancing based on published studies on other primate groups. In accordance with other studies on gibbon cognition (Abordo, 1976; Cunningham, 2006), we further predicted high variability in test performance between individuals but expected overall results comparable to hominids. We expected some individuals of the sample to reach significant success rates under the simpler pointing conditions, while at population level, no significant utilization of any signal type was anticipated.

In addition, observing the gibbons’ grasping behaviour in an OCT opened the possibility to study their expression of hand preferences for this specific task. Although OCTs allow us to investigate manual laterality under controlled settings, this opportunity is only rarely realized (e.g., in Barth, Reaux & Povinelli, 2005). Several papers on hand preferences in hylobatids have been published with almost every single investigation dealing with different situations of hand use. Previous studies focused on preferences for the leading hand during brachiation in one (Stafford, Milliken & Ward, 1990; Redmond & Lamperez, 2004) or several planes of space (Barker, 2008), in feeding (Yuanye, Yunfen & Ziyun, 1988; JE Heestandt, 1987, unpublished data), precision grasping (Olson, Ellis & Nadler, 1990; Christel, 1993) and gesturing (Von Allmen & Geissmann, 2005). Recently, researchers focused on more complex manipulative actions to infer handedness in captive as well as wild gibbons (Morino, 2011; Fan et al., 2017). One of the major findings of these recent investigations was that siamangs (*Symphalangus syndactylus*) show population level left-hand preferences for uni- and bimanual object manipulation (Morino et al., 2017). Comparable data for other gibbon species are still lacking. While routinely executed actions like grasping in the context of locomotion or feeding have been shown to be weakly lateralized in general, actions that require a higher degree of dexterity tend to elicit more strongly lateralized responses among primates (Hopkins et al., 2003; Schweitzer, Bec & Blois-Heulin, 2007). A standardized procedure to infer handedness by means of such manipulative actions in primates is the bimanual tube task (Hopkins, 1995). It requires subjects to manipulate a small tube filled with fruit or vegetable mash to gain the food as a reward. Thereby, one hand is holding the tube while the other one performs the motorically more demanding task of retrieving the food. Statistical evaluation of manipulation attempts later on allows the assessment of hemispheric dominance for manipulative tasks. The usefulness of the tube task for the detection of hemispheric specializations has been validated by neuroanatomical studies (Hopkins & Cantalupo, 2004).

To test if the responses of the gibbons in the OCT are indicative of consistent handedness patterns, we tested them additionally in the tube task. Gibbons routinely exhibit a stabilizing support grip when being stationary, regardless of their posture, leaving only one hand to manipulate objects. We therefore hypothesized that they would exhibit a consistent manual preference for grasping and tube insertions. In accordance with the postural-origins hypothesis (MacNeilage, Studdert-Kennedy & Lindblom, 1987), and based on the available data for siamangs, we expected the left hand to be preferred for these actions. Our study is the first to directly compare hand preferences exhibited by gibbons in bimanual manipulations and unimanual grasping to test for hand preference consistency throughout different situations of hand use.

## Materials and Methods

### Subjects

We studied 25 gibbons housed in four zoological gardens in Germany and France. Of those 25 gibbons (all available individuals at the testing locations) only 11 individuals completed the OCT and 18 individuals finished the tube task resulting in 20 individuals effectively participating in the study (Table 1). The 20 subjects represented six species of hylobatids from the genera *Hylobates*, *Nomascus* and *Symphalangus*. The sample included subjects of all age classes with individuals being between 21 months and putatively over 50 years old at the time of testing. Further details on the identity and life history of the respective individuals are given in Table 1. Eleven individuals participated in the object-choice task, since they successfully passed the priming procedure (see below, Object-choice task, *Apparatus and priming*) and all trials (*Hylobates*: *n* = 3; *Nomascus*: *n* = 7; *Symphalangus*: *n* = 1), and 18 subjects engaged in the tube task (*Hylobates*: *n* = 4; *Nomascus: n* = 13; *Symphalangus*: *n* = 1). Not all gibbons that completed the object-choice task could be included in the tube task sample. The female *H. muelleri* Franziska experienced an arm injury approximately 20 years ago. Since we could not rule out a pertaining influence of this event on her hand preferences, we did not consider her for the sample. The juvenile unnamed male *N. leucogenys* (termed “Juvenile” here) at Duisburg zoo refused to engage in the tube task. All individuals were naive towards the experimenters as well as the experimental conditions.

**Table 1:** Basic information on the identity of subjects included in the study and tests performed (indicated by X) with each subject in the OCT (object-choice task) and/or TT (tube task).

Individual	Species	Sex	Age at testing (years)	Rearing	Locality	OCT	TT
Charlie	*Hylobates lar*	M	>40^a^	Captive	Hamm	X^c^	X
Frodo	*Hylobates lar*	M	21	Captive	Hamm		X
Lissy	*Hylobates lar*	F	37^a^	Wildborn	Rheine		X
Franz	*Hylobates muelleri*	M	>50^a^	Wildborn	Hamm	X^b^^,^^c^	X
Franziska	*Hylobates muelleri*	F	>50^a^	Wildborn	Hamm	X^b^^,^^c^	
Chloé	*Nomascus gabriellae*	F	27	Captive	Mulhouse	X^b^^,^^d^	X
Dakine	*Nomascus gabriellae*	F	9	Captive	Mulhouse	X^c^	X
Dan	*Nomascus gabriellae*	M	25	Captive	Mulhouse		X
Firmine	*Nomascus gabriellae*	F	7	Captive	Mulhouse		X
Connie	*Nomascus leucogenys*	F	27	Captive	Mulhouse		X
Chukhao	*Nomascus leucogenys*	F	10	Captive	Mulhouse	X^c^	X
Lai Cao	*Nomascus leucogenys*	M	1,75	Captive	Mulhouse	X^c^	X
Sophie	*Nomascus leucogenys*	F	44^a^	Wildborn	Duisburg	X^b^^,^^c^	X
Wuki	*Nomascus leucogenys*	F	12	Captive	Duisburg		X
“Juvenile”	*Nomascus leucogenys*	M	3	Captive	Duisburg	X^c^	
Anoie	*Nomascus siki*	F	11	Captive	Mulhouse		X
Chanchi	*Nomascus siki*	M	9	Captive	Mulhouse		X
Dorian	*Nomascus siki*	M	27	Captive	Mulhouse		X
Feng-Shui	*Nomascus siki*	F	7	Captive	Mulhouse	X^d^	X
Jupp	*Symphalangus syndactylus*	M	45^a^	Wildborn	Duisburg	X^b^^,^^c^	X

**Notes.**

Age (in years) refers to the age at the time of testing.

a Age could only tentatively be assigned.

b Individual not consistently showing a support grip during the OCT, allowing for additional analyses on lateralized unimanual grasping (see ‘Materials and Methods’, *Tube task and OCT-hand preference*).

c Female experimenter.

d Male experimenter.

Following the criteria of Call & Tomasello (1996), all of our subjects can be classified as typical “captive” apes, having regular contact to humans related to caretaking but no further notable socialisation influence. In accordance, none of the tested gibbons was hand-reared. Therefore, we do not consider any individual in our sample enculturated. However, OCT tested gibbons of the species *N. leucogenys* and *N. siki* at Mulhouse zoo regularly attended a medical training procedure for approximately 8 months since the start of our study. During this training, usually taking place twice a week for varying time intervals approximating 15 min in total for each individual, they had to respond to a combination of visual and auditory commands, including imperative pointing, to prepare for veterinary interference. Correct responses were positively reinforced. Correspondingly, they were more strongly exposed to human communicative signals than other gibbons kept in zoos. Additionally, regular unobstructed contact with humans during feeding and play sessions was given in the case of the tested siamang at Duisburg zoo.

All subjects voluntarily participated in the respective tasks and were never deprived of water or food during testing. There were no disruptions of the apes’ feeding routine or social environment. All tests were approved by the host zoos visited during the study and adhered to the legal requirements of Germany (Duisburg, Hamm, Rheine) and France (Mulhouse). All experiments complied with the animal testing regulations of the country where they were performed. No ethical permissions were necessary.

### Object-choice task

#### Apparatus and priming

**Figure 1:**
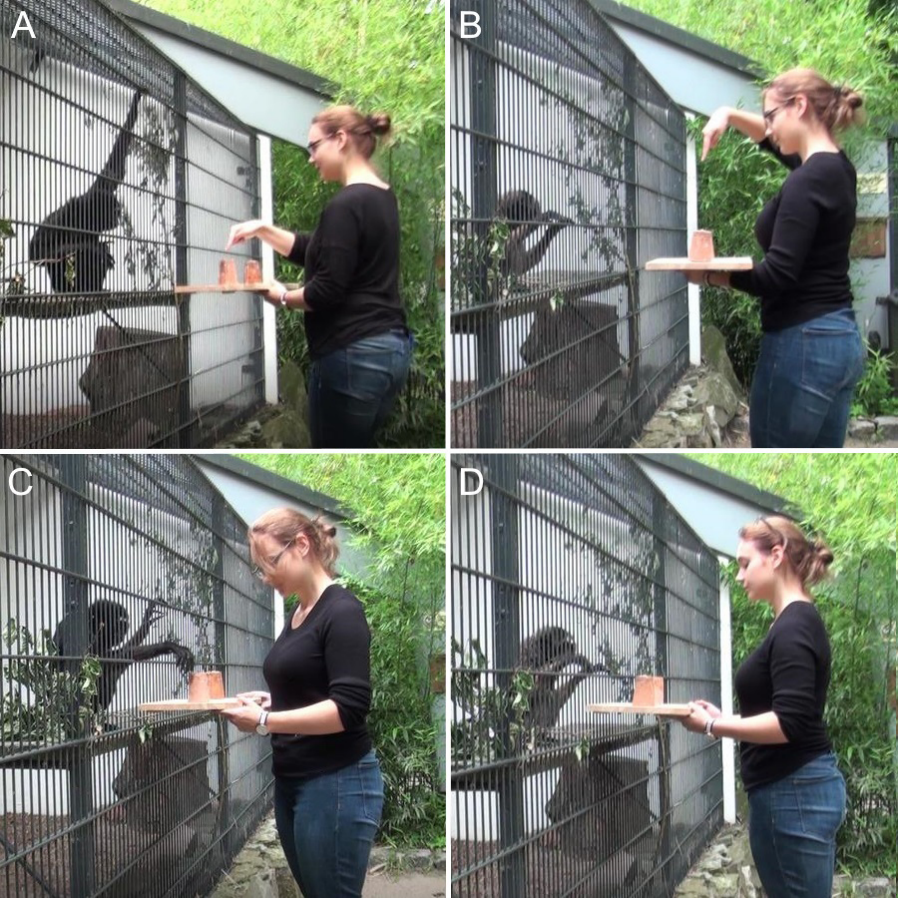
Experimental conditions of the object-choice task illustrated by trials with *H. muelleri*. (A) Near pointing condition. (B) Far pointing condition. (C) Body and gaze orientation condition. (D) Glance condition. Photo credit: Kai R. Caspar.

We performed OCTs following the central object presentation scheme (see Mulcahy & Hedge, 2012). Gibbons were required to choose one of two identical leather cups (7 cm × 15 cm) presented to the apes on a wooden board (30 cm × 45 cm × 2 cm). The cups were positioned on the midline of the board, equidistant to its edges, at a distance of 30 cm from each other. To avoid them to be taken away by the gibbons the cups were fixed to the board using light metal chains. The board was presented from outside of the respective enclosure so that the gibbon had to reach through the wire mesh of the fencing to select a cup (Fig. 1). To elicit initial interest in the test apparatus, food rewards were visibly placed on top of the board and presented to the gibbon. When the ape accepted the food presented on the board, the experimenter visibly baited cups in order to motivate the gibbon to remove the respective cup to gain the reward. After the gibbon repeatedly removed cups subsequently to observing them being baited, the baiting process was relocated behind an opaque barrier. The latter was chosen depending on the surroundings of the apes’ housings. For this, walls or panels in proximity to the enclosure’s fencing were used. As soon as the gibbon independently started to remove cups to access rewards, while unable to observe the baiting process, the trials were counted as valid. Of the 25 gibbons presented with the test apparatus, only 14 eventually actively removed cups with 11 individuals completing 120 trials (see below). All of the latter promptly removed the cups by themselves when presented with the visibly baited apparatus, so that testing could be performed immediately after the apes were initially exposed to the test apparatus.

#### Testing procedure

Raisins, grapes and apple slices were used as food rewards. We did not specifically test for the gibbons’ food preferences but all food types proofed to be highly desirable. The experimenter remained the same for one individual throughout the OCT. Before each trial, one of the cups was baited with a food reward unnoticed by the gibbons. Baiting took place behind opaque barriers as during parts of the priming procedure. A trial was initiated by presenting the baited test apparatus at the fencing while calling the gibbon’s name. If additional motivation was necessary, a food item was offered to get the gibbon’s attention. As soon as the respective individual moved towards the experimenter, a specific referential cue (see below) was given in reference to the baited cup. The experimenter presented the signal to the gibbon at first from a distance of approximately 1.5 m behind the wire mesh, after assuring that the apes’ attention was focused on him-/herself. Subsequently, the experimenter approached the fencing to allow the gibbon to reach for the cups. All cues were given as dynamic-sustained signals and were executed until the gibbon indicated its decision by actively removing one of the cups. By choosing the correct cup, the subject gained the bait as a reward as well as verbal praise. The gibbon retrieved the reward independently from the experimenter. When choosing the incorrect one, the test apparatus was immediately withdrawn from the gibbon, leaving it without the food reward. The location of the reward under the left or right cup was pseudorandomized with a balanced overall left–right ratio. All trials were recorded using a Sony digital video camera recorder (HDR-CX505VE or HDR-CX550VE).

We studied the gibbon’s responses to the visual cues in four signal categories and a control condition corresponding to the protocol of Inoue, Inoue & Itakura (2004). Each condition comprised 24 trials. Therefore, each individual had to complete 120 trials in total. The conditions were as follows:

(1) Near pointing (Fig. 1A): The experimenter points at the baited cup from a short distance of ca. 5 cm with his/her right hand. The index finger is outstretched and the hand of the experimenter is held directly above the respective cup. The head of the experimenter is also oriented although not inclined towards the correct cup while his/her gaze is alternating between the subject and the baited cup.
(2) Far pointing (Fig. 1B): The experimenter points at the baited cup from a distance of 20–30 cm, otherwise the condition is identical to condition 1.
(3) Body and gaze orientation (Fig. 1C): The experimenter is holding the board with both hands and leans towards the baited cup with the upper body portion. The experimenter’s head is inclined towards the baited cup and his/her gaze is fixed on it until the end of the trial.
(4) Glance direction (Fig. 1D): The experimenter is holding the board with both hands and his/her head is facing straight forward. Only the orientation of the experimenter’s eyes indicates the position of the baited cup. The experimenter is glancing at the baited cup until the end of the trial.
(5) Control: The experimenter is holding the board with both hands and his/her head is facing straight forward. No referential cue is given; the experimenter’s gaze is fixed on the gibbon.

All gibbons were free to participate in the object-choice task at any time and were not separated from their conspecifics during testing. Generally, each subject received eight consecutive trials for a specific condition (starting with near pointing) before a new set of eight trials for the next condition (far pointing, followed by body/gaze orientation, glancing, and control) was started. This cycle of 40 trials was repeated three times resulting in 120 trials in total per gibbon with each condition comprising 24 trials (three sets × eight trials). However, since the gibbons were free to leave the experiment at any time and displayed varying motivation to consistently participate in the task, the sets of eight were in parts not finished in a single session and had to be completed later on, sometimes on other test days. Disturbances during a trial by conspecifics could not be prevented completely. Respective trials were not scored, since it could not be excluded that the intervention of other individuals might have altered the decision making of the focus animal. Trials were discarded when a subject other than the focus animal tried to manipulate the testing apparatus or when the latter was approached and/or touched by a conspecific during the test situation. It took subjects between two to seven days of testing to finish all 120 trials (see Table 2).

**Table 2:** Numbers of correct choices in the object-choice task.

Species	Subject	Number of testing days	Control	Near pointing	Far pointing	Body and gaze	Glance	Total X/96
*H. lar*	Charlie	6	9 (*0.307*/0.064)	14 (*0.541*/0.071)	12 (*1*/0.093)	14 (*0.541*/0.071)	10 (*0.541*/0.071)	50 (0.760)
*H. muelleri*	Franz	7	16 (*0.152*/0.057)	**19** **(*0.007*/0.029)**	17 (*0.064*/0.05)	16 (*0.152*/0.057)	13 (*0.839*/0.086)	**66** **(*<0.001*)**
*H. muelleri*	Franziska	7	13 (*0.839*/0.086)	16 (*0.152*/0.057)	**18** **(*0.023*/0.036)**	15 (*0.307*/0.064)	9 (*0.307*/0.064)	58 (0.052)
*N. gabriellae*	Chloé	2	11 (*0.839*/0.086)	16 (*0.152*/0.057)	15 (*0.307*/0.064)	10 (*0.541*/0.071)	16 (*0.152*/0.057)	57 (0.082)
*N. gabriellae*	Dakine	5	15 (*0.307*/0.064)	15 (*0.307*/0.064)	16 (*0.152*/0.057)	11 (*0.839*/0.086)	12 (*1*/0.093)	54 (0.262)
*N. leucogenys*	Chukhao	4	14 (*0.541*/0.071)	15 (*0.307*/0.064)	**18** **(*0.023*/0.036)**	15 (*0.307*/0.064)	17 (*0.064*/0.05)	**65** **(*<0.001*)**
*N. leucogenys*	“Juvenile”	5	8 (*0.152*/0.057)	11 (*0.839*/0.086)	13 (*0.839*/0.086)	10 (*0.541*/0.071)	11 (*0.839*/0.086)	45 (0.610)
*N. leucogenys*	Lai Cao	2	10 (*0.541*/0.071)	16 (*0.152*/0.057)	17 (*0.064*/0.05)	15 (*0.307*/0.064)	13 (*0.839*/0.086)	**61** (0.010)
*N. leucogenys*	Sophie	5	13 (*0.839*/0.086)	15 (*0.307*/0.064)	11 (*0.839*/0.086)	14 (*0.541*/0.071)	15 (*0.307*/0.064)	55 (0.184)
*N. siki*	Feng-Shui	2	12 (*1*/0.093)	**18** **(*0.023*/0.036)**	11 (*0.839*/0.086)	**20** **(*0.002*/0.021)**	11 (*0.839*/0.086)	**60** (0.018)
*S. syndactylus*	Jupp	3	13 (*0.839*/0.086)	**19** **(*0.007*/0.029)**	14 (*0.541*/0.071)	10 (*0.541*/0.071)	10 (*0.541*/0.071)	53 (0.358)
Total X/264			134 (*0.854*/0.093)	**174** **(2.601 × 10^−7^/0.001)**	**163** **(1.632 × 10^−4^/0.014)**	**150** **(*0.031*/0.043)**	137 (*0.580*)	
*H. lar*	Satuki	Unknown	4/8	21	20	23	20	84

**Notes.**

Each subject accomplished 24 trials per test condition (near pointing, far pointing, body and gaze orientation, glance direction) and in the control trials. Group-level data is given as the number of correct choices in all 264 trials (11 individuals × 24 trials) per condition. Two-tailed binomial tests were used to analyze the results. Uncorrected (italics) and adjusted p-values assigned to the respective success rates are given in parentheses for each condition and individual as well as for the group-level data. Bold numbers indicate success rates significantly above chance level according to Benjamini-Hochberg correction (*p* ≤ 0.043). The results of the single subject Satuki tested by Inoue, Inoue & Itakura (2004) are also presented for comparison (this individual performed significantly above chance level under all conditions except for the control).

**Figure 2:**
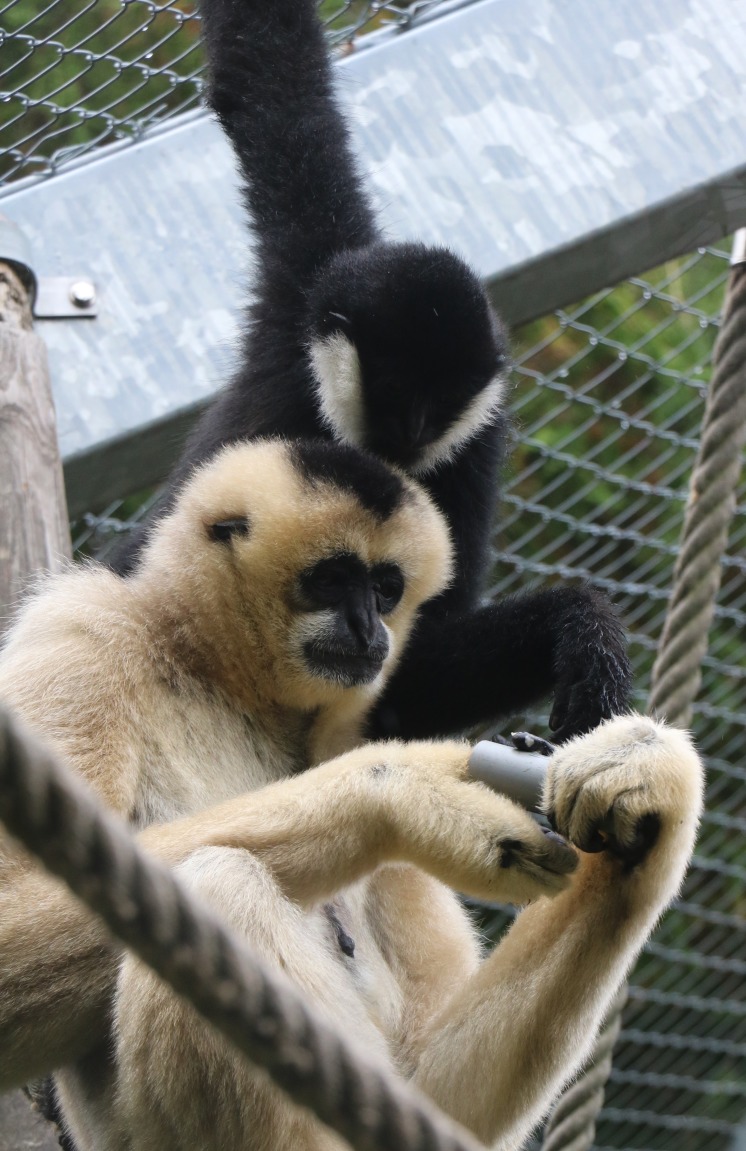
Adult female *N. leucogenys.* engages in the tube task, observed by an infant. Photo credit: Miriam Lindenmeier.

### Tube task

PVC tubes with a length of 10 cm and a diameter of 2.5 cm were used in the tube task. To evoke manipulation by the gibbons, desirable food (mashed bananas, grapes, mashed boiled potato) was smeared on the inside of the tube which was subsequently handed to the subjects through the fencing of the enclosure. For every individual participating in the task, at least 30 insertions into the tube executed within at least six bouts (defined as in Morino et al., 2017) were recorded. Only bimanual insertion events were counted, requiring one hand to stabilize the tube while the other one was inserted to retrieve food (Fig. 2). Foot assisted tube manipulations of any kind were excluded from the analysis. All tests were recorded using a Sony digital video camera recorder (HDR-CX505VE or HDR-CX550VE) and were scored from the videos.

### Data scoring and statistical analysis

The results of the object-choice tasks were scored from the recorded material by a researcher not involved in the testing of the respective subject. In addition to the gibbon’s decision on which cup to select and its success rate under each test condition, we noted the direction from which the ape approached the test apparatus as well as the hand used to remove the cup and the presence or absence of a support grip during each individual trial. For the tube task, we noted the hand used by the experimenter to pass the tube to the animal as well as the fingers used by the gibbon to retrieve the food.

We used two-tailed binomial tests to analyze the results of the OCT for all referential cues as well as for all individuals to determine significant responses above or below chance level and used Benjamini–Hochberg correction (Benjamini & Hochberg, 1995) with a positive false detection rate of 0.1 to address multiple testing (corrected *p*-values are noted as *p*_adjusted_). Given that procedure, uncorrected *p*-values turned out to indicate significance when *p* ≤ 0.043. The Friedman test was applied to evaluate if the success rate of the gibbons changed across trials under a given condition. For this, we compared the success rates of the first eight trials with those of the last eight trials for any given individual and all referential cue types. We set up linear regression models (LM) to identify factors influencing the gibbons’ performance in the OCT. Potential influences (predictors) tested to forecast the outcome of the OCT were attendance in medical training procedures (yes/no), the hand used to select a cup (left/right), hand preference (HI_OCT_) and the direction from which the gibbon approached the board (frontal/sideways).

For the tube task, we created generalized linear mixed models (GLMM), in order to detect factors affecting lateralized responses. We tested the potential influence of age (coded in years), sex, genus, fingers used and the hand used by the experimenter to pass the tube to the gibbon for respective trials. Individuals were used as random effects in the GLMM. The R Packages ‘MuMIn’ and ‘nlme’ (Version 3.4.3, R Core Team, 2017; Bartoń, 2017; Pinheiro et al., 2017) were used to create and evaluate the models. Multi-model inference and model averaging was used to evaluate the tested potential influences. For this, we used the full model average method of Symonds & Moussalli (2011) to calculate the relative Akaike weights of each potential factor. The 95% confidence interval was derived for each potential influence tested. In case the confidence intervals did not include zero, we accepted the respective factors to significantly influence the gibbon’s response in the respective test (OCT or tube task).

For quantifying lateralized responses in both the OCT and the tube task, we calculated the handedness indices (HI) for all individuals in the respective test. For the tube task, we additionally calculated the corresponding binomial *z*-score for each subject tested. HI values were calculated by subtracting the number of left-hand insertions from the number of right-hand insertions which were subsequently divided by the total number of responses (*HI* = (*R* − *L*)∕(*R* + *L*)). Therefore, HI scores could range between −1 (exclusively left-hand insertions) and 1 (exclusively right-hand insertions). Binomial *z*-scores were used to determine the statistical significance of recovered hand preferences in the tube task. Following Hopkins (2013), we rated subjects with *z*-score values higher than 1.96 as right-handed, those with values lower than −1.96 as left-handed, and those with values between −1.0 and 1.0 as ambidextrous. Subjects with *z*-scores within the range −1.96 < *z* ≤  − 1 and 1 ≤ *z* < 1.96 were classified as moderately left- or right-handed, respectively. In most studies, however, (e.g., Morino et al., 2017), individuals falling in these ranges are classified as being ambidextrous or ambiguously handed, too. We tested for population level handedness in *Nomascus* using a one-sample *t*-test. Additional tube task data from this genus were obtained from Fan et al. (2017) and Morino et al. (2017). Pearson’s correlation coefficient was calculated to test for consistent hand use patterns between the object-choice task and the tube task.

**Figure 3:**
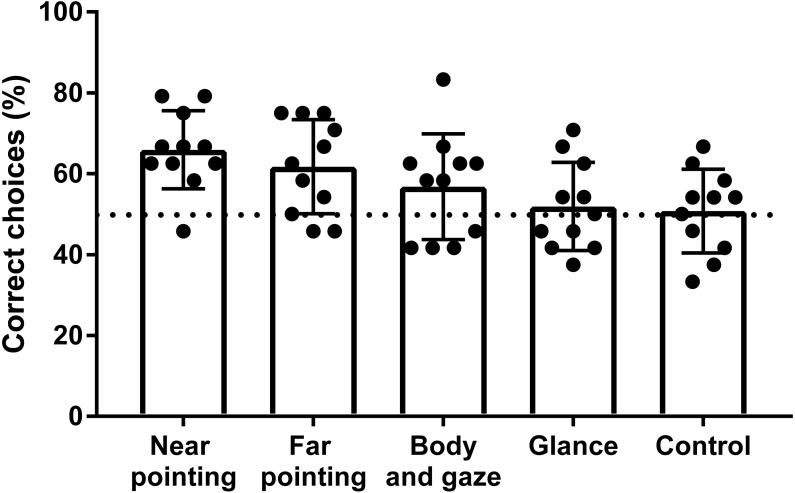
Success rate of hylobatids in the OCT. Bars represent the mean values scored on a group level for each signal category. Points represent the results of individual subjects. The dashed line indicates chance level (50% success rate).

## Results

### Object-choice task

Five of the eleven individuals tested in the OCT performed significantly above chance level in one or more of the signal categories (Table 2). One subject (*N. siki*, Feng-Shui) scored significantly in two signal categories and four others (*H. muelleri*, Franz; *H. muelleri*, Franziska; *N. leucogenys*, Chukhao; *S. syndactylus*, Jupp) in just one so that no single subject successfully made use of all cues given. Four of eleven subjects scored significantly above chance level for all test conditions combined, although one of them (*N. leucogenys,* Lai Cao) did not reach significant results in any single signal category. The highest success rates were found for the pointing gestures (Fig. 3), which both were reliably used to locate rewards at the group level with statistical significance (*p* < 0.001; *p*_adjusted_ < 0.025). The mean success rate of the gibbons when near pointing cues were given was 65.9% with three individuals scoring significantly above chance level. Under the far pointing condition, the mean percentage of correct choices was 61.8% with two subjects selecting correct cups significantly above chance level (Fig. 3). Two other individuals (*H. muelleri*, Franziska; *N. leucogenys*, Lai Cao) scored results indicative of statistical trends close to significance (*p* = 0.064; *p*_adjusted_ = 0.05). Signalling by body orientation and gaze was less well utilized with only one subject (*N. siki*, Feng-Shui) yielding significant results. The mean percentage of correct choices for this signal type was 56.8%. At group level, however, gibbons still showed significant positive responses under the body and gaze condition (*p* = 0.031; *p*_adjusted_ = 0.043). In the glancing condition, the mean percentage of correct choices was 51.0% with no subject responding significantly above chance level. However, one subject (*N. leucogenys* Chukhao) reached results corresponding to a statistical trend approaching significance (*p* = 0.064; *p*_adjusted_ = 0.05). The group level performance in the glancing condition closely matches the one of the control situation. All individuals performed without significant deviations from chance level in the control with the mean success rate being 50.8%.

The OCT-LM suggested the attendance of medical training to be the only tested factor significantly influencing the gibbons’ group level responses in the task (Table 3). It also displayed the highest W evidence calculated of 0.82. There were no significant alterations in the OCT-success rate of the subjects at the individual or group level over time (Friedman test, *p* > 0.05).

**Table 3:** Results of model averaging for generalized linear mixed models describing potential predictors for the tube task results using the Akaike information criterion.

Predictor	Estimate ± SE	2.5% CI	97.5% CI	W evidence
**Medical training**	**0.094 ± 0.042**	**0.011**	**0.176**	**0.82**
Hand used to select	0.031 ± 0.017	−0.002	0.065	0.72
HI_OCT_	0.015 ± 0.035	−0.053	0.083	0.40
Approach direction	0.006 ± 0.034	−0.061	0.072	0.27
Hand used to select*HI_OCT_	0.032 ± 0.032	−0.032	0.095	0.10

**Notes.**

Predictors with a 95% confidence interval not overlapping 0 were accepted as significant and are shown in bold letters.

In total, 70 trials (5%) had to be discarded because of mistakes in procedure made by the experimenter and 9 (0.6%) trials because of interference by conspecifics.

### Tube task and OCT-hand preference

**Figure 4:**
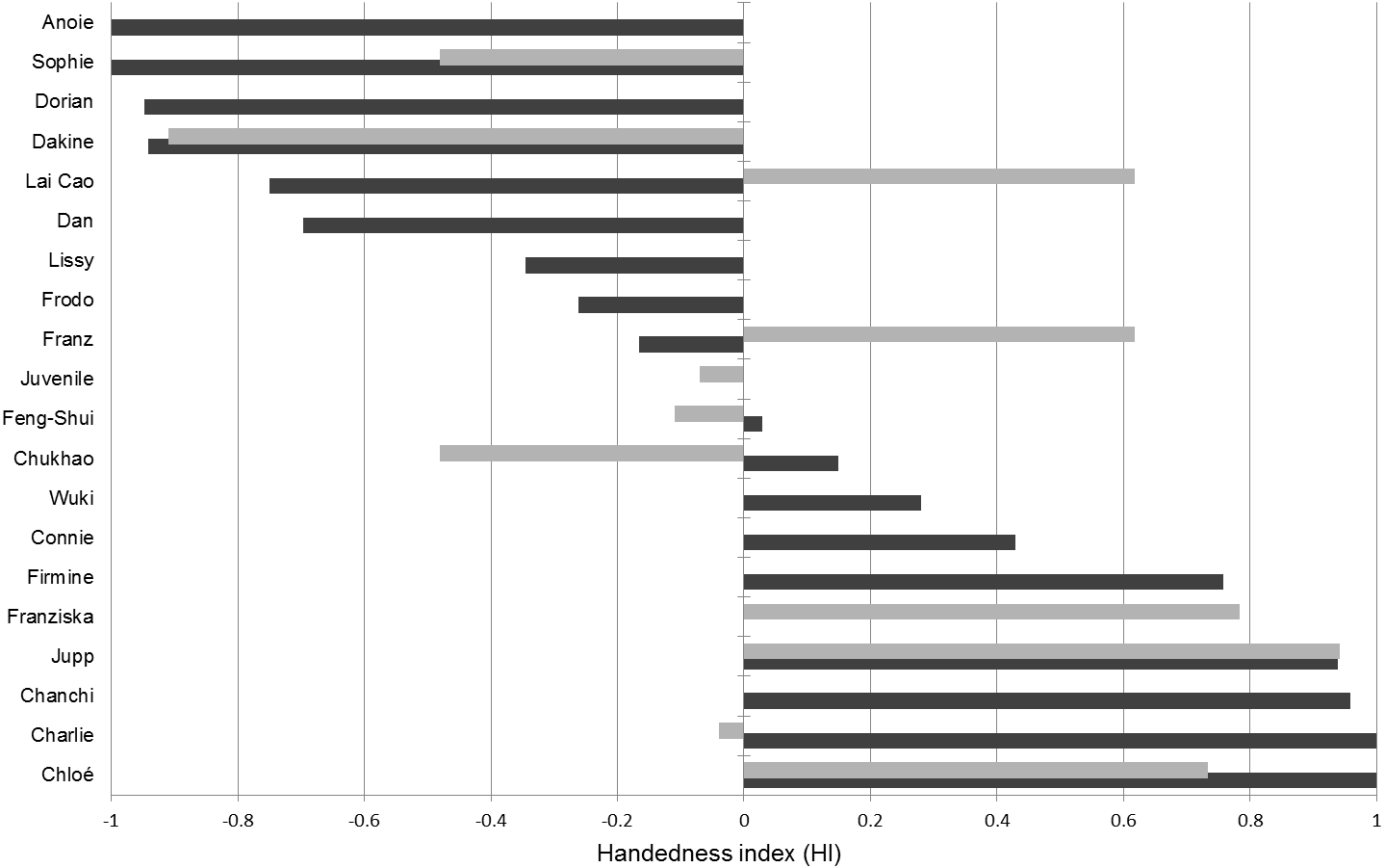
Handedness indices (HI) of hylobatids. Dark bars correspond to HIs established from the tube task (HI_*TT*_) while the light bars correspond to HIs detected in the object-choice task (HI_*OCT*_).

We recorded a total of 962 valid insertions distributed over 433 bouts in the tube task (mean individual results: 24.1 bouts, SD: 22,6; 53.4 insertions, SD: 32,9). The tests revealed that nine of the 18 gibbons in our sample have a pronounced left-hand and six a right-hand preference (Table 4). One subject was classified as ambidextrous and two as moderately right-handed. No overall tendency towards right or left lateralization was found at the group level (Fig. 4). Also, pooling of all available data on tube task performance of *Nomascus* individuals (Table S1) did not reveal a population level hand preference for bimanual manipulation in this genus (*n* = 24; mean HI =  − 0.045; *p* > 0.75).

**Table 4:** Summary of tube task results as well as OCT results regarding the expression of hand preference.

Subject	Species	HI_TT_	Bouts	Inserti-ons	*z*-Score	Two-sided *p*-value	Hand preference
Anoie	*N. s*.	−1	17	52	−7.211	5.55 × 10^−13^	left
Sophie	*N. l*.	−1	31	33	−5.745	9.216 × 10^−9^	left
Dorian	*N. s*.	−0.947	14	76	−8.259	1.47 × 10^−16^	left
Dakine	*N. g*.	−0.941	14	34	−5.488	4.06 × 10^−8^	left
Lai Cao	*N. l*.	−0.75	13	32	−4.243	2.209 × 10^−5^	left
Dan	*N. g*.	−0.697	19	33	−4.004	6.23 × 10^−5^	left
Lissy	*H. l*.	−0.345	17	58	−2.626	0.009	left
Frodo	*H. l*.	−0.262	35	65	−2.109	0.035	left
Franz	*H. m*.	−0.165	109	170	−2.148	0.032	left
Feng-Shui	*N. s*.	0.029	19	68	0.243	0.808	ambidextrous
Chukhao	*N. l*.	0.149	27	47	1.021	0.307	mod. right
Wuki	*N. l*.	0.28	33	36	1.667	0.096	mod. right
Connie	*N. l*.	0.429	23	35	2.535	0.011	right
Firmine	*N. g*.	0.758	13	33	4.352	1.34 × 10^−5^	right
Jupp	*S. s*.	0.939	18	33	5.396	6.799 × 10^−8^	right
Chanchi	*N. s*.	0.9583	9	48	6.639	3.147 × 10^−11^	right
Charlie	*H. l*.	1	8	35	5.916	3.297 × 10^−9^	right
Chloé	*N. g*.	1	14	74	8.602	7.83 × 10^−18^	right

Subject	Species	I_OCT_	*z*-Score	Two-sided *p*-value	Hand preference
Dakine	*N. g*.	−0.91	−10.04	1.0 × 10^−23^	left
Chukhao	*N. l*.	−0.487	−5.477	4.32 × 10^−8^	left
Sophie	*N. l.*	−0.484	−5.295	1.192 × 10^−7^	left
Feng-Shui	*N. s*.	−0.117	−1.278	0.201	mod. left
“Juvenile’ ’	*N. l*.	−0.07	−0.73	0.465	ambidextrous
Charlie	*H. l.*	−0.04	−0.365	0.715	ambidextrous
Franz	*H. m*.	0.617	6.755	1.426 × 10^−11^	right
Lai Cao	*N. l*.	0.617	6.755	1.426 × 10^−11^	right
Chloé	*N. g*.	0.734	8.033	9.491 × 10^−16^	right
Franziska	*H. m*.	0.784	8.581	9.406 × 10^−18^	right
Jupp	*S. s*.	0.942	8.581	9.406 × 10^−18^	right

**Notes.**

TITLE H. l. *Hylobates lar* H. m. *Hylobates muelleri* N. g. *Nomascus gabriellae* N. l. *Nomascus leucogenys* N. s. *Nomascus siki* S. s. *Symphalangus syndactylus* mod. moderately

The tube task-GLMM did not suggest any influence of age, sex, genus or the experimenter’s hand passing the tube on the tube task results (Table 5). There was, however, an effect of digit use (W evidence = 0.83). Accordingly, usage of the thumb, which was the preferred finger to manipulate (Supplemental information, Fig. 1), coincided with stronger left-hand preferences. Hand use frequencies of the left (43.83%) and the right thumb (33.98%) however did not differ significantly (*T* = 0.837; *p* = 0.401). The HI values calculated for those individuals that participated in both tests (tube task and OCT) did not significantly correlate (Pearson *r*^2^ = 0.52, *p* = 0.156; *n* = 9) with five subjects being classified into different hand preference categories based on either OCT or tube task results (Table 4; Fig. 4). Four subjects (indicated in Table 1) did not consistently show a support grip when selecting cups in the OCT, allowing to infer influences of such on the manual lateralization in the task. A significant effect of the presence of support grips on lateralized grasping was found: trials in which a support grip was present exhibited increased manual lateralization in three of the four subjects and the recovered HI values differed significantly from the ones found for trials without one (*p* = 0.013).

**Table 5:** Results of model averaging for logistic regression models describing potential predictors for the object-choice task results using the Akaike information criterion.

Predictor	Estimate ± SE	2.5% CI	97.5% CI	W evidence
**Fingers: Thumb**	−0.459 ± 0.157	**−0.768**	**−0.150**	0.83
Multiple fingers	−0.147 ± 0.153	−0.448	0.153	
Clade: *Nomascus* *Symphalangus*	−0.165 ± 0.437 0.931 ± 0.833	−1.098 −0.847	0.768 2.710	0.43
Sex	−0.008 ± 0.393	−0.842	0.825	0.26
Hand used to pass tube	−0.075 ± 0.055	−0.182	0.033	0.11
Age	−0.001 ± 0.015	−0.033	0.032	0.01

**Notes.**

Predictors with a 95% confidence interval not overlapping 0 were accepted as significant and are shown in bold letters.

## Discussion

### Object-choice task

The gibbons were able to spontaneously utilize different referential cues correctly at the individual as well as at the group level, demonstrating the ability to flexibly adapt to human communicative cues. However, the gibbons’ success in exploiting the pointing gestures could also have resulted from local enhancement effects instead of actual comprehension of the signals. The experimenter’s gesturing hand is in (more or less close) proximity to the correct cup and was probably perceived as a positive food-associated stimulus by the gibbons. Therefore it might have created a local reference point, indirectly guiding the subject’s attention to the correct cup. However, it is difficult to disentangle to which extent the signals were comprehended as directional cues. Local enhancement effects are known to appear in OCTs using peripheral object presentation (Nawroth, Ebersbach & Von Borell, 2014). In OCTs utilizing central object presentation, as presented herein, these effects can be expected to be less severe. The experimenter, the body part giving the cue and the objects to choose from, all are in close proximity to one another and therefore contribute to level out local enhancement effects. Furthermore, the juvenile *H. lar* Satuki observed by Inoue, Inoue & Itakura (2004) performed significantly above chance level when exposed to “body orientation and gaze” and “glance” signals and one of the subjects in this study did so in the former category, too. This demonstrates that hylobatids principally are able to interpret cues which are not prone local enhancement effects. Also, in the OCT performed by Yocom (2010), pointing gestures did not elicit notable success rates in the task. This again calls a primary significance of local enhancement effects in the executed OCT-paradigm into question. Nevertheless, it cannot be excluded that they have affected aspects of the reported findings concerned with pointing gestures.

The overall success rate of the tested gibbons varied profoundly within the sample and at species level. The constant to decreasing success rates of subjects over time argues against notable effects of learning on the outcome of the task. Six individuals performed not above chance level in either of the test conditions. This mirrors the results of Yocom (2010) and likely reflects the original behavioural repertoire of the apes which apparently can be altered as a consequence of prolonged human contact. Similar to great apes, gibbons can adapt to different human communicative cues without specific training.

None of the subjects in our sample reached the success rate of the subject Satuki observed by Inoue, Inoue & Itakura (2004). Differences in experimental design between Inoue, Inoue & Itakura (2004) and our study could be responsible for some of the discrepancies in performance between Satuki and the gibbons tested herein. In the present study, all gibbons had to reach through a fence while Satuki was tested unobstructed. Barriers have been demonstrated to have a negative effect on the OCT-performance of dogs and might similarly influence the gibbons’ responses (Kirchhofer et al., 2012).

Nevertheless, the lower success rates of the subjects in our study compared to Satuki conform to our predictions since this individual must be considered far more socialized among humans than any gibbon involved in the current study. Her remarkable results are best explained as a consequence of behavioural adaptation to human communicative signals based on the available knowledge on great ape enculturation effects. The broad continuum of OCT-success rates displayed by captive gibbons is in accordance with the hypothesis of enculturation effects in the Hylobatidae being similar to hominids. However, the varying OCT-results conform with the already mentioned strong inconsistencies frequently displayed by gibbons in cognitive tasks.

However, there is no indication that the patterns observed in the OCT are the sole consequence of individual predispositions to testing exhibited by the subjects. Instead, the socialization hypothesis is supported by the LM recovering the attendance of medical training as a significant predictor of OCT-success rates. The results of the infant *N. leucogenys* Lai Cao demonstrate that gibbons can learn to interpret human communicative cues by observation from very early age on. Moreover, the results of the two wild caught *H. muelleri* individuals stated to be more than 50 years old at the time of testing show that their responsiveness to the respective signals can be retained to an old age. It should be noted that the unbalanced representation of gibbon species in our small sample could potentially mask phylogenetic influences on the task performance. Since results of other studies suggest that at least at the genus level responses of gibbons in cognitive tests can consistently differ (Cunningham, 2006), we cannot adequately rule out that OCT-performance is not similarly affected.

**Table 6:** Responses of different primate taxa in object choice task-studies providing information on the percental success rate under near pointing conditions.

Taxon	Enculturation	Success-rate	Study
Hylobatidae	No	65.9%	This study
*Hylobates lar*	Yes	87.5%^a^	Inoue, Inoue & Itakura (2004)
*Pan troglodytes*	Mixed	50.2%	Barth, Reaux & Povinelli (2005)
*Pan paniscus, Pan troglodytes, Pongo pygmaeus*	No	58.0%	Mulcahy & Call (2009)
*Pan troglodytes*	No	55.4%	Lyn, Russell & Hopkins (2010)
*Pan troglodytes*	Yes	83.9%	Lyn, Russell & Hopkins (2010)
*Pongo pygmaeus*	Yes	100%^a^	Itakura & Tanaka (1998)
*Macaca mulatta*	No	49.0%^b^	Anderson, Montant & Schmitt (1996)

**Notes.**

All OCTs considered here employed the central object presentation scheme. The given values correspond to the mean success rate of the sample.

a Only one individual was tested.

b Value corresponds to the performance during the first 60 trials.

The gibbons demonstrated successful usage of the near pointing and far pointing gestures as well as, to a lesser extent, the body orientation and gaze cue at the level of the group. No utilization of glance direction was detectable at individual or group level with a success rate mirroring the one of the control condition. Regarding the spontaneous understanding of pointing gestures, the gibbons scored higher at group level than it is usually reported for hominids being housed in zoos or laboratories, which typically do not respond significantly above chance in comparable testing paradigms with central object presentation (see Table 6). The superior performance of the gibbons may be influenced by a sampling bias because only those individuals attended the OCT which were motivated to interact with humans and therefore were potentially more exposed to human communicative signals beforehand. Nevertheless, the OCT-results clearly demonstrate that the three tested gibbon genera are able to utilize pointing gestures and learn to do so without explicit training. Contrary to expectation, several subjects scored higher in the far pointing condition compared to the near pointing one. A possible reason for this might be that the immediate presence of the experimenter’s hand above the correct cup had an unsettling effect on the respective gibbons because it could have conveyed a competitive intention. All individuals showing this pattern avoided direct contact with the experimenter unrelated to food rewards and seemingly retained certain timidity towards humans. The same effect could be assumed for the body orientation condition.

The signal category in which the gibbons were least successful in exploiting cues was glancing without any subject performing significantly above chance level. This result is consistent with earlier studies on non-human primates which showed that the interpretation of such cues is often problematic (Peignot & Anderson, 1999; Barth, Reaux & Povinelli, 2005). Despite this, one individual in our sample (*N. leucogenys*, Chukhao) demonstrated to be able to interpret glance cues correctly to an extent closely approaching significance (*p* = 0.064; *p*_adjusted_ = 0.05). Individual predispositions of Chukhao regarding her responsiveness to the signal or test situation might be considered to explain her specific case. Alternatively, her results might contingently indicate an ability to utilize glance cues. In any case, further studies comparing the performance of gibbons with varying extends of human interaction have to be conducted to elucidate the possible effect of gaze and glance cue enculturation in the Hylobatidae as well as the general informative value of intra- as well as interspecific gaze and glance cues for gibbons. The available data on this topic are rather limited. An early anecdotal report by Bennett (1834) was the first to propose that gibbons, in that case a siamang, are able to infer the attentional state of humans based on gaze direction and to adjust their behaviour accordingly. Contemporary studies on gaze following in gibbons conclude that hylobatids routinely coorient to the gaze of conspecifics as well as humans but lack the more sophisticated visual perspective taking observed in hominids (Horton & Caldwell, 2006; Yocom, 2010; Liebal & Kaminski, 2012). The results by Inoue, Inoue & Itakura (2004) suggested a more proficient use of gaze and glance signals in this primate group, at least in case gibbons have been socialized among humans. Since our study did not examine strongly enculturated hylobatids, we cannot conclude wether Satuki’s results are as exceptional as they appear to be. However, for captive gibbons housed in generic captive environments, we can convincingly disprove a comparable capability to exploit glance cues.

Based on the currently available data it is difficult to retrace the evolutionary history of the ability to utilize gaze and glancing cues. It still appears possible that gibbons share a principal but rarely developed cognitive property to spontaneously interpret gaze and glances as declarative signals with hominids. Beside that option, the ability might have been secondarily lost in gibbons or it could represent an apomorphic character of hominids. To test these assumptions, further data on enculturated gibbons but also other primate species are urgently required. OCT-studies on non-hominoids are scarce and largely restricted to rhesus macaques (*Macaca mulatta*: Anderson, Montant & Schmitt, 1996; Hauser, Glynn & Wood, 2007) and capuchin monkeys (*Sapajus* sp*.*: Anderson, Sallaberry & Barbier, 1995; Itakura & Anderson, 1996). After extensive training, capuchins can learn to use signals conveyed by gaze and glancing (Vick & Anderson, 2000). Apparently, they do not develop this ability through observation only. If enculturated capuchins would also fail in a glancing cue OCT, we could assume that they might not have the capacity to spontaneously interpret this signal type. However, tests like this are still lacking. Remarkably, evidence for the spontaneous comprehension of glance cues in primates other than hominoids was presented for common marmosets (*Callithrix jacchus*) (Burkart & Heschl, 2006) but has seemingly not been replicated, yet. A wider selection of species in and outside of the primate order to study in OCTs is required to retrace the evolutionary history of this specific capacity and how levels of comprehension differ between taxa. Recent results from non-model species (e.g., Hall et al., 2011; Smet & Byrne, 2014) already suggest that the ability to utilize different referential cues in various contexts might be far more common than it was once assumed, at least among mammals.

### Tube task and OCT hand preferences

Hand preference indices established in the OCT and the tube task did not correlate significantly and werethus indicative of inconsistent hand preferences. It should, however, be noted that our sample size was rather small, since only nine individuals participated in both tests. Based on the results of numerous studies, the tube task is established as a reliable indicator of hemispheric specializations and other neuroanatomical correlates of hand preferences (Hopkins & Cantalupo, 2004; Phillips, Sherwood & Lilak, 2007). Deviations from the handedness patterns recovered in the tube task are therefore worthy of remark. The OCT involves motorically simpler actions compared to the tube task which might explain the differences between the handedness patterns. In that case, however, a far lesser degree of lateralization for the OCT compared to the tube task would be expected but was not observed. Although not correlated in direction, both the OCT and the tube task elicited comparably high degrees of lateralization strengths (mean —HI_TT_—: 0.647; mean —HI_OCT_—: 0.526). One reason for the grasping in the OCT being strongly lateralized might be the predominantly suspended posture of the gibbons and the necessity to reach through the fencing. The assumption that posture can be of importance in this context is backed up by the finding that the presence of support grips induced stronger lateralized responses in the OCT. Our results suggest that gibbons show pronouncedly different hand preferences dependent on the hand use situation, a finding consistent with the results of Fan et al. (2017). Nevertheless, the determinants of inconsistent hand preference patterns for varying tasks in hylobatids remain unknown and require further investigation. By comparing our results with those of Von Allmen & Geissmann (2005) who observed five *Nomascus* individuals that were also sampled in the tube task in our study, we can show that a hand preference for manipulative actions does not correspond to the hand use pattern in tactile and imperative gesturing in this genus. While none of the five individuals showed significant degrees of lateralization in gesturing, all of them did so in the tube task. If this finding can be generalized, hylobatids appear to differ from hominids which tend to show lateralized responses in both situations (Hopkins et al., 2005; Prieur et al., 2016a; Prieur et al., 2016b).

Since the three sampled *Nomascus* species diverged less than 2 million years ago (Thinh et al., 2010) and do not show any notable ecomorphological disparities, it is justified to assume a shared handedness pattern for all of them. By combining our tube task results with the data of Fan et al. (2017) and Morino et al. (2017), no hand preference for manipulative tasks at the level of the genus emerged. Obviously, a larger sample size is needed to determine if *Nomascus*, as well as other gibbon species, show left-handedness for bimanual manipulation at the population level in accordance with the postural-origins hypothesis similar to siamangs. Nevertheless, the preliminary data currently available suggest that, at least for *Nomascus* species, this is not the case.

## Conclusions

Our study provides support for the initial claim of Inoue, Inoue & Itakura (2004) that hylobatids are able to use different human communicative cues as referential signals. In addition to that, we also found further evidence for the assumption that enculturation can decisively influence the behavioural repertoire of gibbons. Future studies should further try to characterize gibbons’ responsiveness to different referential signals and the factors influencing their perception in a comparative framework. Similarities as well as differences of gibbon cognition compared to the one of great apes and Old World monkeys could reveal important phylogenetic patterns which may have been overlooked in the past due to model species selection. Additionally, work on larger hylobatid samples could contribute to further clarify the influences of human socialization and also phylogeny on the gibbons’ performance in tests such as OCTs.

With respect to hand preferences, our study suggests that the genus *Nomascus* does not exhibit population level left-handedness for manipulative actions and differs in that respect from siamangs (*Symphalangus*). To retrace handedness evolution among gibbons, extensive sampling of further individuals, especially of the genera *Hoolock* and *Hylobates* will be necessary. Inconsistent handedness patterns across species in the Hylobatidae, a group exhibiting striking anatomical and ecological uniformity, could challenge the traditional postural-origins hypothesis and elucidate general aspects of the evolution of handedness patterns among primates.

##  Supplemental Information

10.7717/peerj.5348/supp-1 Table S1 Compiled data from tube task-studies involving *Nomascus* gibbons used to find population-level biases in lateralization for bimanual tasks

10.7717/peerj.5348/supp-2 Figure S1 Ratios of finger usage observed in the tube task The box plots show that the thumb was the preferred finger to manipulate and was used for 77.81% of insertions.

10.7717/peerj.5348/supp-3 Data S1 Outcome of all trials in the visual cue task

10.7717/peerj.5348/supp-4 Data S2 Outcome of all trials in the tube task

## Additional Information and Declarations

### Data Availability

The following information was supplied regarding data availability: The raw data are available as Supplemental Files.
