# Supplementary material for: Captive gibbons (Hylobatidae) use different referential cues in an object-choice task: insights into lesser ape cognition and manual laterality
Source: PeerJ. 2018 Aug 6;6:e5348. doi: 10.7717/peerj.5348 (PMC6098942; doi:10.7717/peerj.5348)
Supplement: Table S1 [file peerj-06-5348-s001.docx]

| Species | Individual | Sex | Age at time of testing (years) | Age class | Location | HI | Study |
| --- | --- | --- | --- | --- | --- | --- | --- |
| *Nomascus gabriellae* | Chloe | F | 27 | Adult | Mulhouse | 1 | This study |
| *Nomascus gabriellae* | Dakine | F | 9 | Adult | Mulhouse | -0.941 | This study |
| *Nomascus gabriellae* | Dan | M | 25 | Adult | Mulhouse | -0.697 | This study |
| *Nomascus gabriellae* | Firmine | F | 7 | Adult | Mulhouse | 0.758 | This study |
| *Nomascus leucogenys* | Chukhao | F | 10 | Adult | Mulhouse | 0.149 | This study |
| *Nomascus leucogenys* | Connie | F | 27 | Adult | Mulhouse | 0.429 | This study |
| *Nomascus leucogenys* | Lai Cao | M | 21 months | Infant | Mulhouse | -0.75 | This study |
| *Nomascus leucogenys* | Sophie | F | 44 | Adult | Duisburg | -1 | This study |
| *Nomascus leucogenys* | Wuki | F | 10 | Adult | Duisburg | 0.278 | This study |
| *Nomascus leucogenys* | Monjirou | F | ? | Adult | Kagoshima | 1 | Morino et al. 2017 |
| *Nomascus leucogenys* | 93-1 | M | 27 | Adult | Beijing | -1 | Fan et al., 2017 |
| *Nomascus leucogenys* | 95-1 | M | 22 | Adult | Beijing | 0.347 | Fan et al., 2017 |
| *Nomascus leucogenys* | 92-2 | F | 26 | Adult | Beijing | 0.932 | Fan et al., 2017 |
| *Nomascus leucogenys* | Y00-2 | F | 21 | Adult | Beijing | -0.201 | Fan et al., 2017 |
| *Nomascus leucogenys* | _06-2 | F | 10 | Adult | Beijing | -0.139 | Fan et al., 2017 |
| *Nomascus leucogenys* | j10-1 | M | 6 | Subadult | Beijing | -0.585 | Fan et al., 2017 |
| *Nomascus leucogenys* | j2012-3 | M | 3 | Subadult | Beijing | 0.015 | Fan et al., 2017 |
| *Nomascus leucogenys* | _09-2 | F | 6.5 | Subadult | Beijing | 0.127 | Fan et al., 2017 |
| *Nomascus leucogenys* | j2012-2 | F | 3.5 | Subadult | Beijing | 0.195 | Fan et al., 2017 |
| *Nomascus siki* | Anoie | F | 11 | Adult | Mulhouse | -1 | This study |
| *Nomascus siki* | Chanchi | M | 9 | Adult | Mulhouse | 0.958 | This study |
| *Nomascus siki* | Dorian | M | 27 | Adult | Mulhouse | -0.947 | This study |
| *Nomascus siki* | Feng-Shui | F | 7 | Adult | Mulhouse | 0.029 | This study |
