# Supplementary figures and images for: Captive gibbons (Hylobatidae) use different referential cues in an object-choice task: insights into lesser ape cognition and manual laterality
Source: PeerJ. 2018 Aug 6;6:e5348. doi: 10.7717/peerj.5348 (PMC6098942; doi:10.7717/peerj.5348)

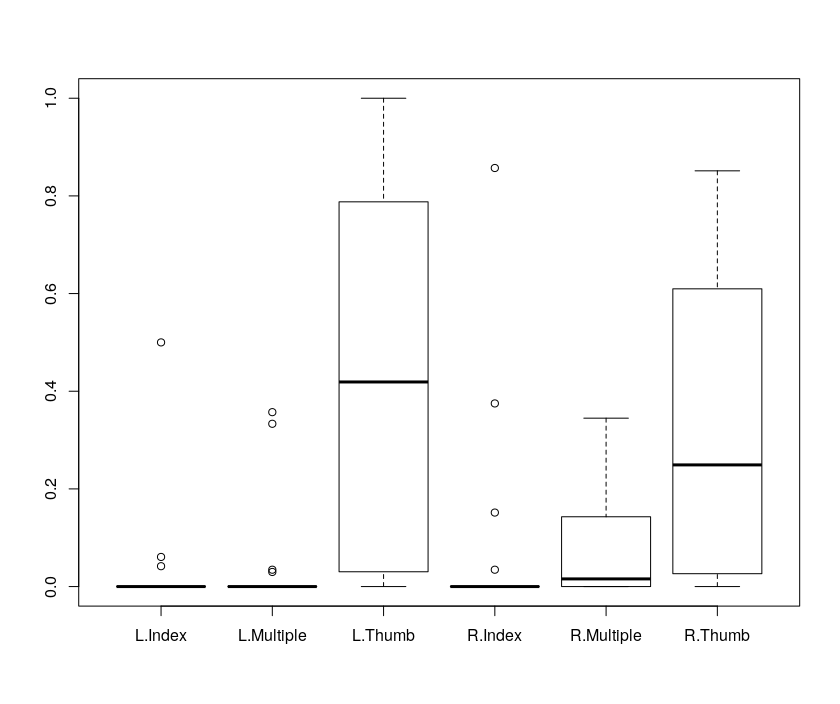

Supplement: Figure S1 — The box plots show that the thumb was the preferred finger to manipulate and was used for 77.81% of insertions. [file peerj-06-5348-s002.png]
